# (Ti,Sn) Solid Solution Based Gas Sensors for New Monitoring of Hydraulic Oil Degradation

**DOI:** 10.3390/ma14030605

**Published:** 2021-01-28

**Authors:** Ambra Fioravanti, Pietro Marani, Giorgio Paolo Massarotti, Stefano Lettieri, Sara Morandi, Maria Cristina Carotta

**Affiliations:** 1 Istituto di Scienze e Tecnologie per l’Energia e la Mobilità Sostenibili (CNR–STEMS), 44124 Ferrara, Italy; pietro.marani@stems.cnr.it; 2 LABSON, Universitat Politecnica de Catalunya, 08222 Terrassa, Spain; giorgio.paolo.massarotti@upc.edu; 3 Istituto di Scienze Applicate e Sistemi Intelligenti “E. Caianiello” (CNR-ISASI), Complesso Universitario di Monte S. Angelo, 80126 Napoli, Italy; stefano.lettieri@isasi.cnr.it; 4 Dipartimento di Chimica, Università di Torino, 10125 Torino, Italy; sara.morandi@unito.it

**Keywords:** (Ti,Sn) solid solution, thick film gas sensors, hydraulic fluid ageing, mineral oil headspace analysis

## Abstract

The proper operation of a fluid power system in terms of efficiency and reliability is directly related to the fluid state; therefore, the monitoring of fluid ageing in real time is fundamental to prevent machine failures. For this aim, an innovative methodology based on fluid vapor analysis through metal oxide (shortened: MOX) gas sensors has been developed. Two apparatuses were designed and realized: (i) a dedicated test bench to fast-age the fluid under controlled conditions; (ii) a laboratory MOX sensor system to test the headspace of the aged fluid samples. To prepare the set of MOX gas sensors suitable to detect the analytes’ concentrations in the fluid headspace, different functional materials were synthesized in the form of nanopowders, characterizing them by electron microscopy and X-ray diffraction. The powders were deposited through screen-printing technology, realizing thick-film gas sensors on which dynamical responses in the presence of the fluid headspace were obtained. It resulted that gas sensors based on solid solution Ti_x_Sn_1–x_O_2_ with x = 0.9 and 0.5 offered the best responses toward the fluid headspace with lower response and recovery times. Furthermore, a decrease in the responses (for all sensors) with fluid ageing was observed.

## 1. Introduction

The hydraulic fluid, in a fluid power system, is generally an oil derived from a mineral source with the addition of some additives [1] chosen according to final ap-plication. It has to be considered as a real extended component interacting with the others [2]. It carries out two crucial tasks: lubrication and power transmission [3] (chapter 2). Therefore, the fluid state is directly connected to the perfect working order of the system. The main reason of hydraulic oil failure is the unavoidable degradation caused by high operational temperature, friction (mechanical stress), contamination by particles, and the presence of water [3] (chapter 15). The degradation of hydraulic oil causes a drop in machine efficiency and reliability. At the same time, it can be an indicator of the state of health of the system; indeed, system breakdowns can cause leakage of pollutant material and a generally expensive unplanned machine stop. For these reasons, the predictive maintenance and, particularly, the real-time monitoring of the oil degradation are crucial [4].

Hydraulic oil is a system described by several chemical-physical properties [3] (acid grade, oxidation level, additives concentration, density, viscosity, dielectric constant, etc.), which change with the oil use and degradation [5].

Usually, the oil analysis is performed on field by means of expensive portable systems or in the laboratory by conventional methods and instrumentation (Karl Fischer method, spectroscopy (spectrometer FTIR), thermal analysis (DSC thermal analyzer), micro-analysis of particulates, analytical ferrography, Brookfield viscometer, etc.). Currently, the conventional methods are standardized, for example, by ASTM (American Society for Testing and Materials), and the state of a hydraulic fluid is described by a set of specific parameters [6].

However, hydraulic oil exhibits a color and a strong and characteristic odor, both of which change with ageing. In fact, it becomes dark with a sour and putrid odor. Starting from empirical observations of the odor change, our novel idea has been to investigate whether a correlation exists between the variation in the oil headspace and the oil ageing by gas sensors based on semiconductor metal oxides. Metal oxide (MOX) semiconductors are multifunctional materials whose optical, conductive, and chemoresistive properties are strongly dependent on defect composition and material morphology [7,8,9,10,11]. Many MOXs are notoriously employed in important environmental and energy applications such as water remediation via the photoinduced oxidation of pollutants [12,13] and hydrogen electrochemical production [14,15], in many cases taking advantages from the nano-composition with advanced functional two-dimensional materials such as MoS_2_ and graphene [16,17,18,19]. However, it is also to be underlined that TiO_2_, SnO_2_, WO_3_, and ZnO also represent the mostly employed active (i.e., gas-sensitive) materials in chemoresistive gas sensing devices [8].

Only few investigations have been published in regard to the topic of monitoring hydraulic oil degradation, including some sensors for on-line lubricant oil monitoring [20], particle counters [21], water sensors in hydraulic oils [22], instruments to analyze the headspace of lubricant oils (as an example, electronic nose or gas chromatograph) [23], MOX sensors to detect diesel in lubricant oils [24], etc. However, to the best of our knowledge, no research work is available that focuses on the correlation between the headspace of hydraulic oil and its ageing detected through thick film gas sensors.

A study of this correlation is the first step for realizing a new online device for oil ageing monitoring, while the use of thick-film technology to prepare MOX gas sensors ensures low cost, small size, easy use, and versatility of the sensor based device [25].

With this aim in view, two new equipment were designed and set-up: (i) a dedicated test bench to age the fluid under controlled conditions; (ii) a laboratory MOX sensor system to test the headspace of the aged fluid samples.

To prepare the set of MOX gas sensors, suitable to detect the analytes’ concentrations in the oil headspace, materials typically used in the fabrication of gas sensors [26,27,28,29,30,31] were initially considered, including TiO_2_, SnO_2_, WO_3_, and ZnO. Concerning ZnO, three different morphologies (nanoparticles aggregates in the form of leaves, long needles, and hexagonal prisms) were considered because the control of the morphology of nanosized metal oxide semiconductors can improve the selectivity toward various gases [32,33]. In addition, a mixed oxide (LaFeO_3_) and (Ti,Sn) solid solutions were synthesized because of their wide potential of combining the advantages and weakening the disadvantages of the single component [34]. Finally, doped oxides such as gold and palladium doped SnO_2_ and tantalum doped TiO_2_ have been prepared.

All the functional materials, synthesized in the form of nanopowders, were characterized by X-ray diffraction (XRD) and scanning electron microscopy (SEM) to analyze the crystalline structure and the morphology.

The nanopowders were subsequently deposited by means of screen-printing technology, to obtain thick film MOX gas sensors. Measurements of dynamical responses toward the fluid headspace were carried out. It resulted that sensors based on (Ti,Sn) solid solution offered the best responses toward the fluid headspace analytes with lower response and recovery times. Furthermore, a decrease in the sensors responses with fluid ageing was observed.

## 2. Materials and Methods

### 2.1. Hydraulic Oil and Its Ageing

**Figure 1:**
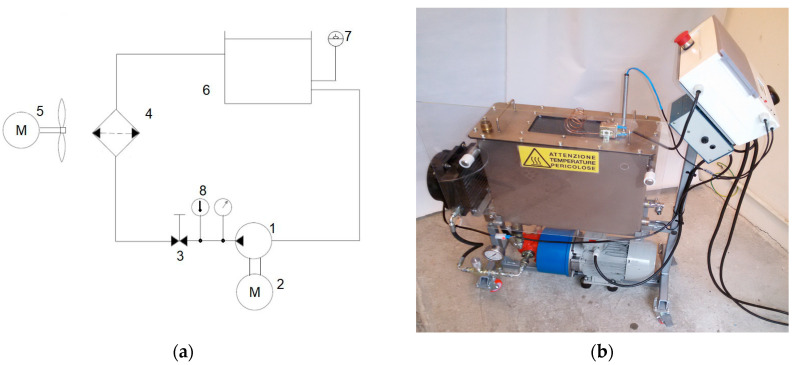
Scheme (**a**) and image (**b**) of hydraulic test bench realized to age the hydraulic oil.

A specific hydraulic oil, JOHN DEERE–HY-GARD JDM J20C (Deere & Company, Moline, Illinois, USA), derived from a mineral source has been chosen among the fluids used for lubrication and hydraulic power in agricultural tractors and compatible with application requiring the following viscosity grades SAE 5W-30, 10W-30 o ISO VG46-VG68 [35]. A John Deere agricultural tractor (John Deere 6170 M), which uses this oil type, has been taken into account to determine the number of working hours after which a complete oil change is necessary. As reported in the correspondent operating manual, a complete oil change corresponds to 1500 working hours. Hence, the ageing and sampling of the oil have been performed from 0 (fresh oil) to 3000 h (steps of 250 h) to study the oil property modifications as a consequence of ageing. A hydraulic test bench, used to age the selected fresh oil, was realized to perform a continuous and rapid oil ageing, under controlled conditions and excluding contamination by external agents. The schematic layout (according to ISO 1219 standard) and the image of the developed oil ageing test bench are shown in Figure 1a,b, respectively. It is composed by the main components below: a pump (1) driven by an electric motor (2), a throttle valve (3), an oil cooler (4) an electric fan drive (5), a 30 L reservoir (6), a fluid level sensor (7), a temperature sensor (8) and an electronic control unit. During the working of the test bench, the fluid is stressed and heated by the pump operation and the throttling of the relief valve. The electronic control provides the heat exchanger switching on/off to maintain the oil temperature at about 90 °C.

### 2.2. Array of MOX Gas Sensors

A dedicated set of MOX gas sensors was prepared starting from the synthesis of semiconductor nanopowders through the wet chemistry method using reagent-grade starting materials as received by the Merck Group supplier (Milan, Italy). All prepared oxides, with notes about their synthesis process, are summarized in Table 1. Additional details are reported in the related references of previously published works. In Table 1, the shorter name of the oxides is also listed as they will be used hereinafter.

The morphology and the crystalline structure of the obtained powders were analyzed with a Carl Zeiss Sigma scanning electron microscope (Carl Zeiss, Oberkochen, Germany) and a Philips PW 1830 vertical diffractometer in Bregg–Brentano geometry (PANalytical, formerly Philips Analytical, Almelo, the Netherland) (Cu Kα radiation, 40 kV, 30 mA) provided with a graphite monochromator along the diffracted beam. Diffraction patterns were collected over the range 10–120° (2θ) with steps of 0.02° and 10 s of dwell time. The parameters of the unit cells were estimated by using the FullProf program (release 2011) [43] through which a Rietveld analysis (structure profile refinement) was performed. Scherrer’s formula was used to calculate the average crystallite size [34].

To realize the MOX gas sensors, viscous pastes were first prepared by adding to the synthesized nanopowders an organic vehicle and a small amount of bonding agent that has the function of promoting the film adhesion to the substrate. The thick films of about 20 μm were deposited by means of a serigraphic printer (Aurel, Italy) onto miniaturized alumina substrates, each one with an area of 2.5 × 2.5 mm^2^ and a thickness of 0.25 mm. On the substrate front side, interdigitated gold contacts are present, and on the back side, a platinum heater element. Finally, the films were thermally treated by exposing them to a firing process at 650 °C for 1 h in a muffle furnace. For us, this the temperature corresponds to a good compromise between the requested sintering and a moderate grains coalescence. More details and descriptions about thick film technology can be found in [44].

### 2.3. Laboratory MOX Sensors System

**Figure 2:**
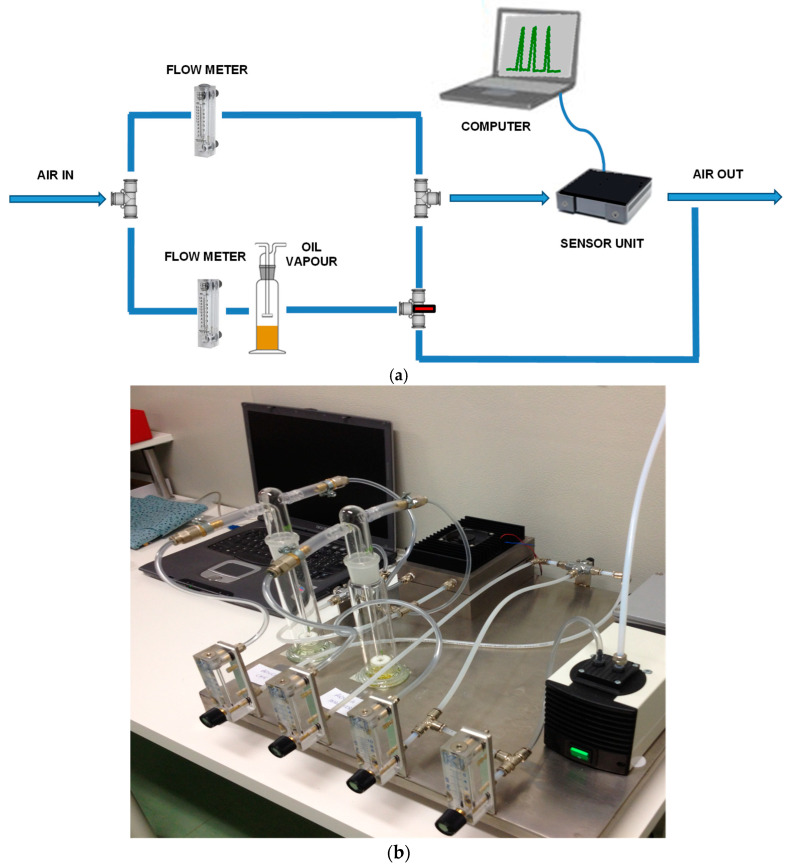
Scheme (**a**) and image (**b**) of laboratory MOX sensors system set-up to examine the aged hydraulic oil samples.

To study the oil ageing, we expressly realized a system based on an array of nanostructured semiconductor oxide gas sensors. Its scheme and its image are shown in Figure 2a,b, respectively.

A sealed chamber lodging up to three MOX sensors, a temperature sensor, and a humidity sensor constitute the core of the sensors monitoring unit. It also includes an electronic circuitry for each sensor, the main electronic control unit, the firmware, and the software developed by using Labview to record the electrical signals of the sensors. To complete the system, a pneumatic circuit managed by mass flow controls and gas taps is included to collect from Drechsel bottles (250 mL) and carry the oil vapor through the sensor box.

The electrical characterization of the sensing layers was carried out by using the flow-through technique. The measurement of conductance was performed while maintaining a flow rate of 0.5 L/min using synthetic air as the carrier gas in dry conditions and at room temperature. Electrical measurements were at least performed on three units of each kind of sensing film. The sensing films’ dynamical responses toward the mixture of air and volatile compounds in the oil headspace were obtained at different sensors’ working temperatures (300, 350, 400, 450, and 500 °C). The sensor response to oil vapor is defined as the ratio between the conductance in the presence of the volatile compound mixture and the conductance in air. The response and recovery times were defined as the time required to reach 90% of the total resistance change in adsorption and desorption [45].

## 3. Results and Discussion

### 3.1. Hydraulic Oil Ageing Samples

**Figure 3:**
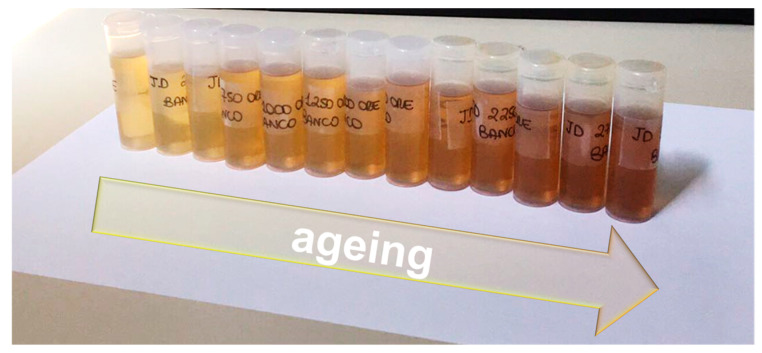
Sequence of oil samples aged from 0 to 3000 h (steps of 250 h) through the hydraulic test bench.

The fresh oil was aged using the dedicated hydraulic test bench with no interruptions and the samples were collected from 0 to 3000 h (steps of 250 h). In Figure 3, the complete series of oil samples clearly shows that with ageing, the oil color changes, becoming progressively darker as the working hours increase.

### 3.2. Characterizations of Sensors’ Materials

XRD patterns of all synthesized samples confirm a pure single phase with the exceptions of TTV and T-Ta oxides and an average crystallite size range from 4 to 87 nm. Crystalline phases and space groups, obtained from Rietveld refinement, as well as average crystallite sizes, calculated by Scherrer’s formula, are summarized and reported in Table 2.

It is worth noting that all (Ti,Sn) solid solutions, as synthesized, show a rutile phase (the most stable TiO_2_ phase) with very small crystallite sizes. Pure TiO_2_ is subjected to crystalline phase transition from anatase to rutile that usually occurs between the temperatures of 600 °C and 700 °C, producing exaggerated grains growth [46,47]. Therefore, the direct synthesis of TiO_2_ nanomaterials with a chemical composition close to titania in rutile form is certainly advantageous to maintain the small particles’ dimension. Indeed, grain size reduction is extremely important for gas sensors based on metal oxides as it enhances the surface–volume ratio [48].

**Figure 4:**
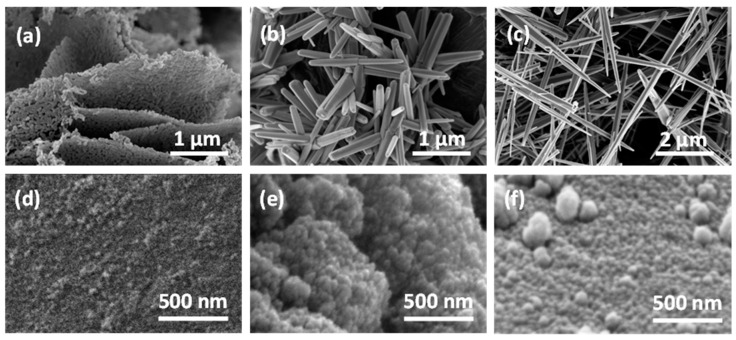
SEM micrographs of ZnO leaves (**a**), ZnO prisms (**b**), and ZnO needles (**c**), ST50 (**d**), ST70 (**e**), and ST90 (**f**).

SEM observations of all oxide powders showed a homogeneous distribution in particle size and shape. For the sake of brevity, in this section, all powder morphologies are described, while some of them are shown in Figure 4, as an example.

ZL (Figure 4a) is constituted by porous rhombic leaves in which the fine structure highlights the presence of spherical roundish nanoparticles with a grain size in the range of 40–60 nm. ZP (Figure 4b) and ZN (Figure 4c) are made of elongated crystals; hexagonal prisms of about 2 μm long, 50–150 nm wide and needles about 5 μm long and 200–300 nm wide , respectively.

The other powders are constituted by spherical particles with grain size of nanometric dimension. In particular, the following show grain dimension ranges:

- SnO_2_, SnO_2__Au, and SnO_2__Pd from 20 to 40 nm agglomerated in a roundish structure of about 100–200 nm,
- STN from 10 to 30 nm,
- TiO_2_ from 20 to 40 nm agglomerated in a roundish structure of about 50–100 nm,
- TTV from 20 to 40 nm,
- T-Ta from 30 to 60 nm,
- WO_3_ from 60 to 90 nm, and
- LaFeO_3_ from 50 to 80 nm agglomerated in a hexagonal structure of about 1 μm.

Concerning the (Ti,Sn) solid solutions, in agreement with the crystallite size value, SEM analysis shows, for each stoichiometry (0.1 ≤ x ≤ 0.9), morphologies with very small grain sizes (up to 30 nm) also for x = 0.9. In Figure 4, the SEM micrographs of ST50 (Figure 4d), ST70 (Figure 4e), and ST90 (Figure 4f) are shown.

### 3.3. Laboratory MOX Sensors System: Oil Ageing Characterization

With the aim to find one or more suited MOX sensors and the best system working conditions to detect the concentrations of analytes in the fluid headspace during its ageing, a wide series of systematic measurements was carried out.

All the tests were performed at room temperature. As it will be shown below, already at room temperature, the analytes’ concentrations in oil vapor were clearly detectable by some of the prepared sensors. This result is of crucial importance for the future application in the fluid power system.

The first investigations, which were necessary to select the best system working condition, the MOX sensors, and their best working temperature, were conducted using only two oil samples: 0 h (fresh oil) and 3000 h of ageing. Electrical measurements were carried out while maintaining a constant flow rate of 0.5 L/min and by choosing a flow ratio between the carrier gas (synthetic air) and the volatile compounds mixture of 1:1, i.e., the best with respect to those tested of 2:1 and 1:2. The volume of oil samples used for the primary tests was 3 mL. The dynamical responses of the sensing films (three units of each kind) were obtained in the presence of a mixture of air and volatile compounds in the oil headspace of 0 and 3000 h samples at different sensor working temperatures (from 300 to 500 °C in steps of 50 °C) in order to: (i) Define the best working temperature for each sensor type, (ii) study the different responses toward oil headspaces, and (iii) select the sensors able to detect the variation in analytes’ concentrations in aged oil from the fresh one.

The resulting best working temperatures are summarized in Table 3. All the dynamical responses toward the oil vapors are ascribable to reducing gases, but only few sensors were able to detect appreciable differences between the headspace of fresh and aged oil. Indeed, in order to perform a complete scan of the oil samples at different aging and detect when the analytes’ concentration in the headspace changes, a minimum difference is needed between the two responses. The criteria chosen to the sensors’ selection is based on the ratio between the response to oil headspace of the 0 h sample (R_0h_) and the response to oil headspace of the 3000 h one (R_3000h_). The responses ratio for each sensor is reported in Table 3 and only sensors that offer a R_0h_ / R_3000h_ ≥ 2 have been used to test the complete series of oil samples. They are TS5, TS9, and ZL sensors.

**Figure 5:**
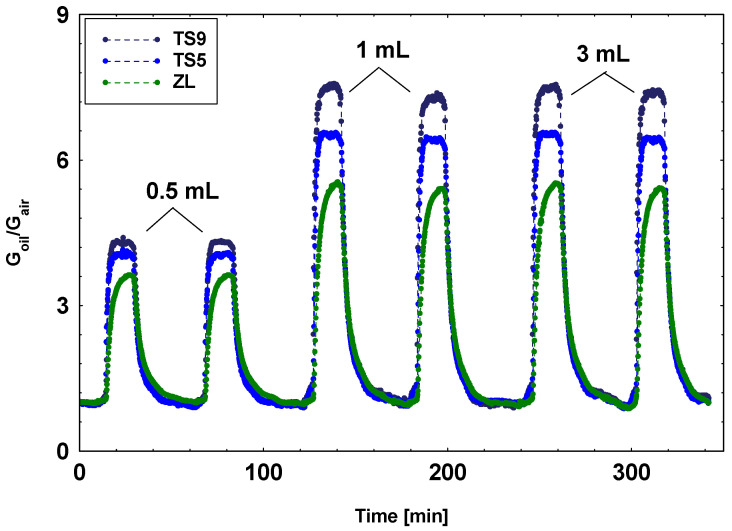
Dynamic responses toward fresh oil samples of 0.5, 1, and 3 mL volume for TS9, TS5, and ZL, respectively.

To study the relation between the sensor response and the oil volume in Drechsel bottles, a specific test on the oil sample volume was performed. The responses of the three selected sensors toward 0.5, 1, 2, 3, and 5 mL of fresh oil samples were compared. The responses to 0.5 mL samples resulted lowest. In addition, no significant variations were shown among the 1, 2, 3, and 5 mL samples responses. As examples, in Figure 5, the dynamical responses toward fresh oil samples of 0.5, 1, and 3 mL volume are reported. The present result attests that oil samples with a volume of 1 mL are of sufficient volume to obtain the best responses with respect to the geometry of Drechsel bottles used in this study. A volume of 1 mL corresponds to the minimum quantity for creating a homogeneous oil layer that completely covers the base of the bottles. Therefore, there is not a volume that maximizes the responses but a minimum quantity below which the responses decrease.

**Figure 6:**
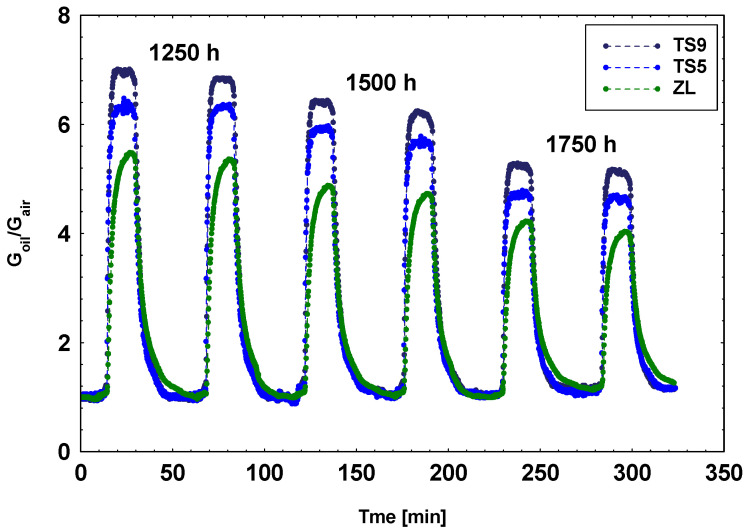
Dynamic responses of three MOX sensors toward the oil samples aged 1250, 1500, and 1750 h.

In Figure 6, the dynamic responses of the three selected MOX sensors toward the oil samples aged 1250, 1500, and 1750 h are shown.

**Figure 7:**
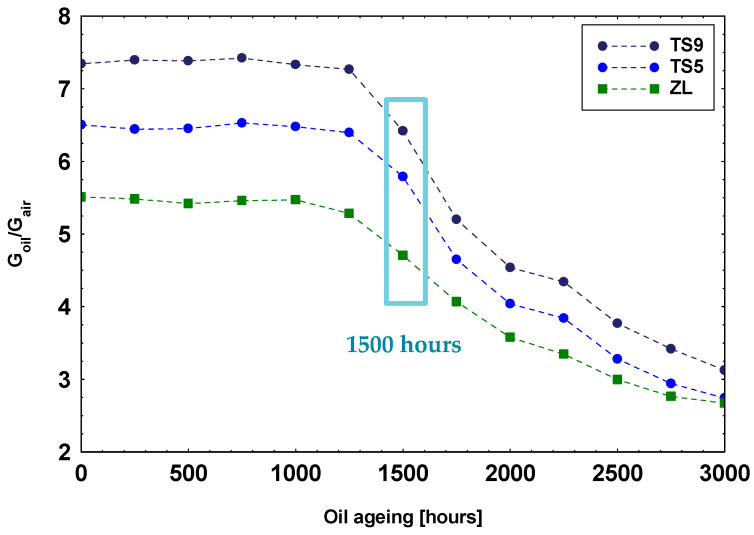
Responses of the complete series of oil samples aged from 0 to 3000 h (steps of 250 h) for the three selected sensors.

In Figure 7, are summarized the average sensors’ responses toward the complete series of oil headspaces corresponding to samples aged from 0 to 3000 h with steps of 250 h. The responses toward headspaces of samples from 0 to 1250 h of ageing are similar but not identical for each sensor. We have interpreted this result not as a change of compound concentrations in the oil headspaces but rather to variations in the environmental conditions to which the whole measuring system is subjected. At about 1500 h of ageing, a rapid decrease in sensors’ response occurred, suggesting a progressive reduction in volatile compounds concentration. This present result is in agreement with oil specifications reported in the operating manual of the considered John Deere agricultural tractor, which fixed the complete oil substitution at 1500 working hours.

Other important detector parameters to design a device with optimum performances are the response and recovery times. In this study, they were evaluated for TS9, TS5, and ZL sensors on the response curves of Figure 5 (on 1 mL sample), summarized in Table 4.

It turned out that the TS9 and TS5 sensors offer lower response (about 1 min) and recovery (about 10 min) times with respect to ZL sensors. However, the sensor time parameters are dependent on the measuring system and not only by the sensor itself. Indeed, as previously proven [49], the reduction in the sensor’s volume chamber results in a decrease in the sensor response and recovery times.

## 4. Conclusions

The aim of the present study was to find a correlation between the variation in the oil headspace and the oil ageing by using MOX gas sensors in order to realize a new low-cost, small size, easy-to-use, and online device for oil ageing monitoring. Until now, this topic is not covered by the literature, but it can provide a new solution for the predictive maintenance and prevent breakdown in hydraulic machines, a direct consequence of the hydraulic oil degradation.

Two new equipment have been successfully designed and set-up: (i) a dedicated test bench to age the fluid under controlled conditions; (ii) a laboratory MOX sensor system to test the headspace of the aged fluid samples. A specific hydraulic oil derived from a mineral source has been chosen and aged for 3000 h. Among the prepared MOX gas sensors, those based on solutions Ti_x_Sn_1−x_O_2_ with x = 0.9 and 0.5 Sn and ZnO leaves exhibited the best responses toward the headspace analytes of the examined oil type sensors, and they were able to detect the variation in analytes’ concentrations in oil at different ages. TS9 also exhibited the lowest response and recovery times.

Furthermore, a decrease in the sensors’ responses with fluid ageing was observed starting from 1500 h of ageing, suggesting a decrease in headspace analytes’ concentrations.

Therefore, a correlation was established between the hydraulic oil aging and the composition of their vapors. The possibility of monitoring the hydraulic oil ageing by using MOX gas sensors was also demonstrated. This novel methodology combined with thick-film technology used to realize the MOX sensors can offer the perfect solution to develop a real-time and online oil sensor. Investigation will be aimed at validating this methodology by testing sensors based on different oxides and performing additional characterizations of the oil aged samples, also for other kind of hydraulic fluids.

## Figures and Tables

**Table 1:** Summary of all prepared oxides with notes about their synthesis process.

Sample Name	Oxide	Reagents, Medium, and Catalyst	Calcination Process	Ref.
T	TiO_2_	Ti(IV) n-butoxide, hydroalcoholic media	2 h at 450 °C	[34]
T-Ta	TiO_2__10%atTa	Titanium(IV) isopropoxide, Tantalum(V) ethoxide in hydroalcoholic media	2 h at 400 °C	[36]
TTV	Ti:Ta:V 100:15:5	Ti(IV) n-butoxidein hydroalcoholic media	2 h at 400 °C	[37]
S	SnO_2_	Tin(II)2-ethylexanoate, hydroalcoholic media, and diluted HNO_3_	2 h at 550 °C	[34]
S-Au	SnO_2__0.4 wt.%-Au	SnO_2_ nanopowders, Gold(III) bromide, in water solution	3 h at 120 °C ^1^	[38]
S-Pd	SnO_2__0.4 wt.%-Pd	SnO_2_ nanopowders, Palladium(II) nitrate hydrate, in water solution	3 h at 120 °C ^1^	[38]
TS1, TS3, TS5, TS7, TS9	Ti_x_Sn_1-x_O_2_ x = 0.1, 0.3, 0.5, 0.7, 0.9	Ti(IV) n-butoxide, Tin(II)2-ethylexanoate, hydroalcoholic media, and diluted HNO_3_	2 h at 550 °C	[34]
STN	Sn:Ti:Nb 100:42:5	Tin(II)2-ethylexanoate, Ti(IV) n-butoxide, Niobium(V) bromide, hydroalcoholic media, and diluted HNO_3_	2 h at 550 °C	[39]
W	WO_3_	Tungsten hexachloride, alcoholic media, and 2,4-pentanedione	2 h at 650 °C	[40]
ZL	ZnO leaves	Zinc nitrate hexahydrate, water, and ammonia solution	2 h at 450 °C	[41]
ZN	ZnO needles	Zinc nitrate hexahydrate, water, and ammonia solution	not necessary	[41]
ZP	ZnO prisms	Zinc nitrate hexahydrate, water, and HMTA	not necessary	[41]
LF	LaFeO_3_	Potassium hexacyanoferrate(III), Lanthanum nitrate, and water solution	30 min at 700 °C	[42]

^1^ The doped SnO_2_ has been obtained by the impregnation method. Hence, the thermal treatment is not a calcination, but it corresponds to a drying process.

**Table 2:** Crystalline phases, space groups, and crystallite sizes of all prepared materials after calcination evaluated from XRD patterns.

Oxide	Crystalline Phase	Space Group	Crystallite Size (nm)
TiO_2_	Anatase 100%	I4_1_/amd	12.0
T-Ta	Anatase (Rutile traces)	I4_1_/amd	15.0
TTV	Anatase ≈ 70% Rutile ≈ 30%	I4_1_/amd P4_2_/mnm	20.0
SnO_2_, SnO_2__Au, SnO_2__Pd	Rutile	P4_2_/mnm	10.6
Ti_x_Sn_1–x_O_2_ x = 0.1, 0.3, 0.5, 0.7, and 0.9	Rutile	P4_2_/mnm	7.8, 4.4, 4.4, 6.3, 11.7
STN	Rutile	P4_2_/mnm	21.0
WO_3_	Monoclinic pseudo-cubic	P12_1_/n1	87.0
ZnO leaves	Hexagonal wurtzite	P6_3_mc	26.0
ZnO needles	Hexagonal wurtzite	P6_3_mc	36.0
ZnO prisms	Hexagonal wurtzite	P6_3_mc	46.0
LaFeO_3_	Orthorhombic Perovskite	Pbnm	48.0

**Table 3:** Best working temperature, response ratio R_0h_ / R_3000h_ for all tested sensors, and the selected sensors.

Sensor Type	Best Working Temperature (°C)	R_0h_/R_3000h_	Selected Sensor (R_0h_/R_3000h_ ≥ 2)
T	450	1.2	-
T-Ta	450	1.2	-
TTV	400	1.4	-
S	450	1.0	-
S-Au	450	1.0	-
S-Pd	450	1.0	-
TS1	500	1.0	-
TS3	500	1.1	-
TS5	500	2.4	X
TS7	500	1.6	-
TS9	500	2.4	X
STN	500	1.0	-
W	450	1.2	-
ZL	400	2.0	X
ZN	450	1.6	-
ZP	450	1.8	-
LF	350	1.1	-

**Table 4:** Response and recovery times for TS9, TS5, and ZL sensors.

Sensor Type	Response Time (min)	Recovery Time (min)
TS9	1.0	9.3
TS5	1.4	10.3
ZL	3.0	13.3

## Data Availability

All data are included in the paper.
